# Autophagy Regulation Using Multimodal Chlorin e6-Loaded Polysilsesquioxane Nanoparticles to Improve Photodynamic Therapy

**DOI:** 10.3390/pharmaceutics15051548

**Published:** 2023-05-20

**Authors:** Hemapriyadarshini Vadarevu, Adeola Julian Sorinolu, Mariya Munir, Juan L. Vivero-Escoto

**Affiliations:** 1 Department of Chemistry, The University of North Carolina at Charlotte, Charlotte, NC 28223, USA; hvadarev@uncc.edu; 2 Nanoscale Science Program, The University of North Carolina at Charlotte, Charlotte, NC 28223, USA; 3 Civil and Environmental Engineering Department, The University of North Carolina at Charlotte, Charlotte, NC 28223, USA; asorinol@uncc.edu (A.J.S.); mmunir@uncc.edu (M.M.)

**Keywords:** photodynamic therapy, autophagy, polysilsesquioxane nanoparticles, combine therapy, codelivery

## Abstract

Photodynamic therapy (PDT) is a promising anticancer noninvasive technique that relies on the generation of reactive oxygen species (ROS). Unfortunately, PDT still has many limitations, including the resistance developed by cancer cells to the cytotoxic effect of ROS. Autophagy, which is a stress response mechanism, has been reported as a cellular pathway that reduces cell death following PDT. Recent studies have demonstrated that PDT in combination with other therapies can eliminate anticancer resistance. However, combination therapy is usually challenged by the differences in the pharmacokinetics of the drugs. Nanomaterials are excellent delivery systems for the efficient codelivery of two or more therapeutic agents. In this work, we report on the use of polysilsesquioxane (PSilQ) nanoparticles for the codelivery of chlorin-e6 (Ce6) and an autophagy inhibitor for early- or late-stage autophagy. Our results, obtained from a reactive oxygen species (ROS) generation assay and apoptosis and autophagy flux analyses, demonstrate that the reduced autophagy flux mediated by the combination approach afforded an increase in the phototherapeutic efficacy of Ce6-PSilQ nanoparticles. We envision that the promising results in the use of multimodal Ce6-PSilQ material as a codelivery system against cancer pave the way for its future application with other clinically relevant combinations.

## 1. Introduction

Photodynamic therapy (PDT) is a minimally invasive localized treatment modality that has emerged as an alternative or supplementary approach to chemotherapy and surgery. PDT has been clinically available and approved to treat cancers such as head and neck cancer, non-small-cell lung cancer, prostate cancer, and colon cancer [1,2]. PDT involves three major components, namely photosensitizer (PS), light, and oxygen. During PDT, photoactivated PSs transfer energy to surrounding molecular oxygen in the cells to generate highly reactive singlet oxygen (^1^O_2_) (type II reaction). In addition, PSs can transfer electrons to generate short-lived PS radical species (type I reaction) that subsequently produce a range of compounds called reactive oxygen species (ROS), such as superoxide anions and hydroxyl radicals [3,4]. An overload of ROS causes significant toxicity by oxidative stress, which eventually leads to cell death. Apoptosis has been reported as the primary regulated cell death mechanism in PDT [5,6]. Furthermore, an iron-dependent cell death mechanism called ferroptosis, which is characterized by extreme cellular lipid peroxidation, can also be triggered by PDT [7,8,9,10].

In recent years, autophagy has been studied as another cellular mechanism that impacts the PDT outcome. Autophagy is a stress response program that is a consequence of PDT independent of cell death signaling [11]. Autophagy is a process of degrading and renewing cytoplasmic components, which is upregulated under cellular stress conditions such as protein aggregate accumulation, infection, and oxidative stress [12,13]. The mechanisms of resistance for PDT include increased expression of antioxidant genes and other protective programs such as autophagy. Autophagy is discussed both as a cytoprotective response and, in some cases, a feature of cell death following PDT, which depends on sub-cellular localization of the PS, type of ROS, and target cell characteristics [14]. The process of autophagy involves the clustering of ubiquitin-positive proteins into larger structures that become enclosed with autophagosomes and subsequently degraded within lysosomes after fusion. Autophagy relies on the formation of double-membraned vesicles known as autophagosomes, leading to the degradation of their cargo, such as damaged proteins or organelles, promoted by autophagy-related (Atg) proteins [15]. Autophagy is activated in response to external stimuli, such as hypoxia, starvation, and therapy, in cancer cells and is, therefore, often considered as an adaptive and pro-survival mechanism. By this means, the accumulation of misfolded proteins is avoided, leading to prolonged survival after PDT [16]. Therefore, PDT efficacy can be improved by combination approaches involving modalities that target these resistant pathways [17,18].

A wide variety of nanocarriers have been employed to enhance the PDT effect by increasing the stability and targeting ability of PSs [19,20,21,22]. In addition, nanoparticle-based formulations that combine PSs with other therapeutic agents have recently been reported [23]. Polysilsesquioxane (PSilQ) NPs are a class of hybrid silica nanoparticles formed by crosslinking condensation of functionalized trialkoxysilanes, affording high loading capacity of the functional moiety and thus providing an interesting platform for therapeutic loading and delivery [24,25,26]. PSilQ NPs have been utilized to improve the PDT effect against different types of cancers. The use of this platform for PDT treatment of cancer has been demonstrated in vitro and in vivo [10,27,28,29,30,31]. Herein, we hypothesize that PSilQ NPs can be designed to carry both a PS agent and an autophagy inhibitor to efficiently reduce the resistant pathways related to this cellular mechanism to finally improve the PDT effect. To inhibit autophagy, two strategies were pursued in this work; first, we used the pharmacological late-stage autophagy inhibitor and metal-chelating agent di-2-pyridylketone 4,4-dimethyl-3-thiosemicarbazone (Dp44mT) [32]. As a second option, we utilize siRNA that suppresses the synthesis of p62/SQSTM1 autophagosome cargo protein, which plays an important role in the early stages of autophagy as it links the ubiquitin-positive cargo material to the Atg8 family proteins in the nascent phagophore membrane [33].

**Scheme 1:**
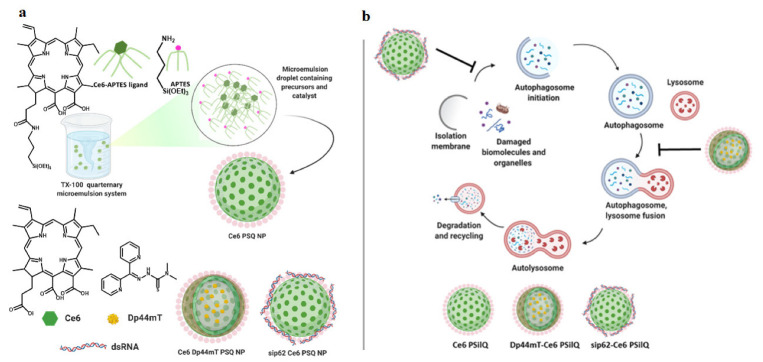
(**a**) Schematic representation of the synthesis of Dp44mT-Ce6-PSilQ and sip62-Ce6-PSilQ NPs. (**b**) Cellular process of autophagy and proposed interference mechanisms using PSilQ nanoparticles: sip62-Ce6-PSilQ nanoparticles target the early autophagosomal formation and sequestration stage. Meanwhile, Dp44mT-Ce6-PSilQ nanoparticles target the autophagosome–lysosome fusion stage. Created with BioRender.com https://www.biorender.com/ (accessed on 10 March 2022).

In this work, we synthesized and characterized PSilQ NPs containing Chlorin e6 (Ce6), which is a second-generation PS agent widely used for the PDT of cancer (Ce6-PSilQ NPs) [34,35]. In addition, we fabricated and characterized Ce6-PSilQ NPs to address both autophagy inhibitory approaches, one loaded with Dp44mT inhibitor (Dp44mT-Ce6-PSilQ) and another one carrying siRNA that targets p62/SQSTM1 (sip62-Ce6-PSilQ NPs) (Scheme 1). The phototherapeutic performance of these materials was evaluated in vitro using HT29 colon cancer cells. PDT has been widely explored for the treatment of colon cancer [36]. Our results show that autophagy is an outcome of PDT-induced oxidative stress prominently for nanoparticle formulations of PSs localized in lysosomes. Dp44mT-Ce6-PSilQ NPs prematurely terminated autophagy by blocking the fusion of autophagosomes with lysosomes, with a consequent accumulation of autophagosomes that resulted in an enhancement of apoptosis. In the case of the inhibition of the p62/SQSTM1 protein, the combination sip62-Ce6-PSilQ NPs did not yield the same result in enhancing PDT. The results suggest that inhibition of autophagy flux but not inhibition of proteins involved in autophagosomal sequestration boosts apoptosis after PDT by Ce6-PSilQ NPs.

## 2. Materials and Methods

### 2.1. Synthesis of Ce6-PSilQ NPs

To fabricate Ce6-PSilQ NPs, first the Ce6 silane ligand was prepared according to the following protocol: 5.9 µmol (3.5 mg) of Ce6 was added into 1.4 mL of dichloromethane (DCM). To this solution, 35.3 µmol (6.8 mg) of 1-ethyl-3-(3-dimethylaminopropyl) carbodiimide (EDC) hydrochloride solution dissolved in 0.4 mL DMSO was added. The flask was then placed in an ice bath and stirred for 10 min. To this mixture, 35.3 µmol (4.1 mg) of N-hydroxysuccinimide (NHS) dissolved in 0.7 mL DMSO was added to the flask and was kept at room temperature for 3 h. A diluted ethanolic solution in water (3 mL, EtOH:H_2_O/25:75% *v*/*v*) was added to the flask to afford precipitation of the product (Ce6-SE). The silane derivative Ce6-TES was prepared by adding 45 µL of APTES (215 µmol) and 7 µL of TEA (73 µmol) to 12 mg (16.2 µmol) of Ce6-SE dissolved in 2 mL of aqueous phase (DMSO:H_2_O/80:20% *v*/*v*). The final solution was stirred for 1 h. This mixture was used as-prepared for the fabrication of the nanoparticles.

The synthesis of Ce6-PSilQ NPs was carried out through a reverse microemulsion method [27]. The organic phase of the reverse microemulsion system was prepared by mixing Triton X-100 (1.8 g, 1.7 mL), 1-hexanol (1.6 mL), and cyclohexane (7.5 mL). The “in situ” prepared Ce6-TES reaction mixture was directly added to the organic phase under vigorous stirring (350 rpm) and room temperature. After the addition of the precursors, 100 µL of NH_4_OH (25% *w*/*w*) was diluted to 10% *v*/*v* in water and was added to the microemulsion system. The mixture was dialyzed against ethanol to destabilize the microemulsion, and the formed nanoparticles were separated from the solution by centrifugation. The nanoparticles were washed twice with ethanol to remove unreacted reagents, and the final product was stored in the same solvent.

### 2.2. Synthesis of Dp44mT-Ce6-PSilQ NPs

The organic phase of the reverse microemulsion system was prepared by mixing Triton X-100 (1.8 g, 1.7 mL), 1-hexanol (1.6 mL), and cyclohexane (7.5 mL). The as-prepared Ce6-TES solution and Dp44mT (0.8 mg, 2.7 µmol) were dissolved in 0.2 mL of aqueous phase (DMSO:H_2_O/80:20% *v*/*v*). This mixture was added to the organic phase under vigorous stirring (350 rpm) and room temperature. After the addition of the precursors, 100 µL of NH_4_OH (25% *w*/*w*) was diluted to 10% *v*/*v* in water and was added to the microemulsion system. The mixture was dialyzed against ethanol to destabilize the microemulsion, and the as-made nanoparticles were separated from the solution by centrifugation. The nanoparticles were washed twice with ethanol to remove unreacted reagents, and the final product was stored in the same solvent. A summary of the synthesis process is illustrated in Scheme 1a.

### 2.3. Synthesis of siRNA-Ce6-PSilQ NPs

siRNA duplexes (Santa Cruz Bio, CA, USA) were dissolved in nuclease-free cell culture grade water at a concentration of 10 µM and stored at −20 °C. The concentrated 10 µM stock of siRNA was further diluted to 800 nM in siRNA dilution buffer (Santa Cruz Bio, CA, USA). A 100 µL suspension of Ce6-PSilQ NPs (200 µM Ce6) was prepared in serum and antibiotic-free DMEM. To this Ce6-PSilQ NP stock solution, 100 µL of 800 nM siRNA stock was dispensed and mixed well by pipetting. The final solution was incubated at room temperature for 30 min and spun down at 13,000 rpm for 15 min after incubation. The supernatant with any non-bonded siRNA was discarded. The nanoparticles were washed once with 200 µL of 50:50 *v*/*v* mixture of serum and antibiotic-free DMEM and siRNA dilution buffer and subsequently centrifuged at 13,000 rpm for 15 min. The resulting siRNA-Ce6-PSilQ NPs were resuspended in 200 µL of serum and antibiotic-free DMEM.

### 2.4. Cellular Uptake and Intracellular Localization of FITC-Conjugated siRNA-Ce6-PSilQ NPs

HT29 cells were cultured at a density of 20,000 cells per well in a 24-well plate containing 500 µL of medium and maintained for 24 h at 37 °C with 5% CO_2_ in a humidified incubator. Cells were then treated with Ce6-PSilQ NPs (0.5 mL) at concentrations equivalent to 5 and 10 µM of Ce6 and incubated for 12–14 h at 5% CO_2_ atmosphere at 37 °C. Afterwards, the cells were washed with phosphate buffer, followed by detachment of cells using 0.25% trypsin-EDTA. The cells were then suspended in DBPS for analysis with the flow cytometer (BD LSR ™ cell analyzer) using PE-Cy7-A channel. Ce6-PSilQ NPs equivalent to 10 µM of Ce6 were complexed with 40 nM FITC-conjugated control siRNA to give FITC-labeled siRNA^FITC^-Ce6-PSilQ NPs. HT29 cells were seeded at 30,000 cells per well in a 24-well plate containing 500 µL of complete DMEM and maintained for 24 h in a humidified incubator at 37 °C with 5% CO_2_. Cells were then treated with siRNA^FITC^-Ce6-PSilQ NPs at Ce6 concentrations equivalent to 5 and 10 µM suspended in serum and antibiotic-free DMEM and incubated for 24 h at 5% CO_2_ atmosphere at 37 °C. Following treatment, the cells were washed with DPBS twice and harvested using trypsin. Cells were resuspended in DPBS for flow cytometry analysis using PE-Cy7-A and FITC channels for Ce6 and siRNA^FITC^, respectively. For confocal laser scanning microscopy, HT29 cells at a density of 100,000 cells per well were seeded onto a coverslip, placed in 6-well plates, and incubated at 37 °C with 5% CO_2_ atmosphere to allow adhesion. After an incubation time of 24 h, the cells were treated with siRNA^FITC^-Ce6-PSilQ at a fixed concentration of 10 µM in 2 mL of serum and antibiotic-free media for a period of 12–14 h. Cells were rinsed three times with cold PBS, and nuclei were stained with Hoechst 33342 for 15 min at 37 °C in a humified incubator. After an additional rinse with PBS, the coverslips were mounted onto the glass slides with media and imaged using an Olympus Fluoview (Tokyo, Japan) FV 1000 confocal laser scanning microscope.

### 2.5. Phototoxicity Assessment

The MTS cell proliferation assay was used to determine cell viability. HT29 cells (3000 cells per well) were seeded into 96-well plates and incubated overnight at 37 °C. After that, the cells were treated with various concentrations of Ce6-PSilQ NPs or free Ce6 ranging from 1 to 5 µM of Ce6 and incubated for 24 h at 37 °C. To analyze cell viability with the combined treatment of PDT and autophagy inhibitor, Dp44mT-Ce6-PSilQ NPs or a 1:6 molar mixture of free Ce6 and Dp44mT were added at concentrations ranging from 1 to 20 µM based on Ce6. The cells were incubated for 24 h at 37 °C. To evaluate the PDT in combination with siRNA-induced gene silencing, siNeg-Ce6-PSilQ NPs or sip62-Ce6-PSilQ NPs were added at Ce6 concentrations ranging from 1 to 20 µM of Ce6 and 4 to 80 nM of siRNA. The treated cells were incubated for 24 h at 37 °C. Next, cells were washed once with cold DPBS followed by irradiation with red light (633 nm, 25 mW/cm^2^) for 20 min in cold DPBS. The solution was aspirated, and cells were resuspended in complete DMEM and incubated at 37 °C for an additional 24 h. Subsequently, cells were incubated at 37 °C with 20% *v*/*v* of the CellTiter 96^®^ MTS solution in complete DMEM for 4 h. In parallel, one unirradiated duplicate plate was maintained for each treatment condition to serve as a control (dark) experiment. Finally, the optical density value of each well at 490 nm was measured using a Multiskan microplate reader. Cell viability (%) was calculated by analyzing absorbance values recorded at 490 nm using a microplate reader. Cell viability (%) was calculated as follows: viability = (A_sample_ − A_blank_)/(A_control_ − A_blank_) × 100%, where A_sample_, A_control_, and A_blank_ denote absorbance values of the sample, control, and blank wells. The IC_50_ values were determined using GraphPad Prism (v8.3.0 for Windows, La Jolla, CA, USA), fitting the normalized viability data to a nonlinear regression.

### 2.6. Measurement of Intracellular ROS Level

HT29 cells were seeded in 6-well plates at a density of 50,000 cells per well and incubated at 37 °C for 24 h. After that, the cells were treated with Ce6-PSilQ NPs or free Ce6 at concentrations equivalent to 2.4 µM Ce6 or Dp44mT-Ce6-PSilQ or a mixture of Dp44mT:Ce6 (1:6 molar) at concentrations equivalent to 2.4 µM Ce6 and 0.4 µM Dp44mT. The cells were incubated for 24 h at 37 °C. Next, cells were washed once with cold DPBS and incubated in serum-free media containing 10 µM DCFDA for 30 min at 37 °C in the dark. Then, cells were washed twice with DPBS and irradiated with red light (630 nm, 25 mW/cm^2^) for 20 min in cold DPBS. Cells were collected by trypsinization and resuspended in DPBS before reading out the ROS-positive population by flow cytometry (BD Fortessa). In parallel, an unirradiated duplicate plate was maintained for each treatment condition to serve as a control (dark) experiment. DCFDA-stained blank cells were seeded in each plate and used to determine the background fluorescence.

### 2.7. Autophagy Assessment by Flow Cytometry

HT29 cells were seeded in 6-well plates at a density of 50,000 cells per well and incubated at 37 °C for 24 h. After that, the cells were treated with Ce6-PSilQ NPs or free Ce6 at concentrations equivalent to 2.4 µM Ce6 or Dp44mT-Ce6-PSilQ NPs or a mixture of Dp44mT:Ce6 (1:6 molar) at concentrations equivalent to 2.4 µM Ce6 and 0.4 µM Dp44mT. HT29 cells were cultured in serum-free media to mimic serum starvation and promote macro autophagy. Serum-starved cells treated with 10 µM chloroquine were used as a positive control group. The cells were incubated for 24 h at 37 °C. The cells were subsequently infected with the RFP-GFP-LC3 baculoviral (ThermoFisher Scientific, Waltham, MA, USA) constructs using a concentration of 30 viral particles per cell. The infected cells were incubated for 16 h at 37 °C and 5% CO_2_. Subsequently, the cells were irradiated using red light (630 nm, 25 mW/cm^2^) for 20 min at room temperature after washing twice with DPBS. RFP-positive populations and GFP-positive populations were read out by flow cytometry (BD Fortessa, Piscataway, NJ, USA) 6 h after irradiation.

### 2.8. Autophagy Assessment by Confocal Microscopy

HT29 cells were seeded in a 6-well plate with glass coverslips at a density of 25,000 cells per well and incubated at 37 °C for 24 h. After that, the cells were treated with Ce6-PSilQ NPs or Dp44mT- Ce6-PSilQ NPs at concentrations equivalent to 1.2 µM Ce6 and incubated for 24 h at 37 °C. The cells were subsequently infected with the RFP-GFP-LC3 baculoviral (ThermoFisher Scientific, Waltham, MA, USA) constructs using a concentration of 30 viral particles per cell. The infected cells were incubated for 16 h at 37 °C and 5% CO_2_. Subsequently, the cells were irradiated using red light (630 nm, 25 mW/cm^2^) for 20 min at room temperature after washing twice with DPBS. Cells were replenished with media for 6 h, and after that, the glass coverslips were washed once with DBPS and mounted onto glass slides for imaging autophagic vesicles by confocal microscopy (Olympus Fluoview, Tokyo, Japan).

### 2.9. In Vitro Analysis of Cell Apoptosis and Necrosis

Annexin V-FITC with live/dead co-staining was used for the assessment of cell apoptosis. HT29 cells were seeded into 6-well plates at a density of 50,000 cells per well and incubated overnight at 37 °C. After that, the cells were treated with various concentrations of Ce6-PSilQ NPs or free Ce6 at concentrations equivalent to 2.4 µM Ce6 and incubated for 24 h at 37 °C. To determine the apoptotic effect caused by the combined treatment of PDT and autophagy inhibitor, Dp44mT-Ce6-PSilQ NPs or a mixture of Ce6:Dp44mT (1:6 molar) at concentrations equivalent to 2.4 µM Ce6 and 0.4 µM Dp44mT were added. The inoculated cells were incubated for 24 h at 37 °C. Next, cells were washed once with cold DPBS followed by irradiation with red light (633 nm, 25 mW/cm^2^) for 20 min in cold DPBS. The solution was removed and complete DMEM was added to the cells, which were incubated at 37 °C for an additional 24 h. Subsequently, the cells were washed once with DPBS and once with binding buffer (1X, BD biosciences). After that, the cells were suspended in binding buffer, followed by the addition of 5 µL of Annexin V-FITC staining solution for 15 min, and then washed once with the binding buffer (0.1X). Afterwards, 5 µL SYTOX Blue (Thermo Fisher Scientific) staining solution was added for 5 min. Finally, the percentage of apoptotic cells was determined using flow cytometry directly after SYTOX Blue staining without an intermediate wash step.

The cell apoptosis assessment for PDT in combination with sip62 was carried out using the following materials: siNeg-Ce6-PSilQ NPs or sip62-Ce6-PSilQ NPs at concentrations equivalent to 2.4 µM and 4.8 µM of Ce6 and 9.6 nM and 19.2 nM of sip62 duplexes. The cells were inoculated with the materials and incubated for 24 h at 37 °C. Next, cells were washed once with cold DPBS followed by irradiation with red light (633 nm, 25 mW/cm^2^) for 20 min in cold DPBS. The solution was removed, and complete DMEM was added to the cells, which were incubated at 37 °C for an additional 24 h. The cells were treated following the protocol described above to determine the percentage of apoptotic cells.

### 2.10. Relative mRNA Expression

The knockdown of p62/SQSTM1 expression was evaluated by quantitative PCR. HT29 cells were grown in 6-well plates at a seeding density of 100,000 cells per well. The cells were maintained for 24 h at 37 °C and 5% CO_2_ atmosphere. The cells were then treated for 24 h with Ce6-PSilQ NPs or siNeg-Ce6-PSilQ NPs or sip62-Ce6-PSilQ NPs at concentrations of 2.4 µM Ce6 and 9.6 nM siRNA. The cells were rinsed once with PBS, detached from the tissue culture treated surface using cell dissociation buffer (Thermo Fisher Scientific) and collected via centrifugation. The isolation and purification of RNA from cells was carried out using a Purelink RNA mini kit along with in column DNase I treatment. Purified RNA was eluted in nuclease-free water and stored at −20 °C. The cDNA synthesis was performed using an iScript cDNA synthesis kit (1708890, Bio-Rad, Hercules, CA, USA). The extracted RNA was mixed with iScript reaction mix, iScript reverse transcriptase, and nuclease-free water as per the manufacturer’s protocol. The complete reaction mix was incubated in a thermal cycler programmed with the following protocol: 5 min priming at 25 °C, 20 min reverse transcription at 46 °C, and 1 min RT inactivation at 95 °C. The contents were held at 4 °C. After the cDNA synthesis, RT-PCR was performed using SYBR Green Supermix (Bio-Rad) and a CFX96 Real-Time System (Bio-Rad). A 20 µL solution was prepared using 10 µL of Universal SYBR Green Supermix, 1 µL of PrimePCR primer pair mix (Biorad), 100 ng of cDNA, and nuclease-free water. The solution was then subjected to the following protocol: an initial step of 95 °C for 30 s for polymerase activation and DNA denaturation, followed by 35X cycles of 95 °C for 15 s, 60 °C for 30 s, and a fluorescence reading. The mRNA expression relative to untreated cells was then quantified in the Bio-Rad CFX Manager software (Bio-Rad, Hercules, CA, USA) using the ΔΔCq method with a GAPDH reference.

### 2.11. Statistics

All the experimental results in the manuscript are reported as mean ± standard deviation (SD) unless mentioned otherwise. For the analysis of nanoparticle size using TEM, 50 nanoparticles were evaluated using Image J. The hydrodynamic size, ξ-potential, and Kaiser’s ninhydrin test were carried out in triplicates or more. The amount of Ce6 and Dp44mT loaded was analyzed in triplicates using three different batches of nanoparticles. Cellular uptake, apoptosis, and autophagy assays were evaluated using flow cytometry with a minimum of 10,000 singlets and quantified in triplicates. For the cell viability studies, GraphPad Prism was used to calculate the IC_50_ values (*n* = 6). Statistical analysis was performed by one-way ANOVA using Tukey’s multiple comparison test. All the statistical analysis was performed using GraphPad Prism (v8.2.0), with a *p*-value < 0.05 considered to be statistically significant.

## 3. Results

### 3.1. Synthesis and Physicochemical Characterization of Ce6-PSilQ NPs

**Figure 1:**
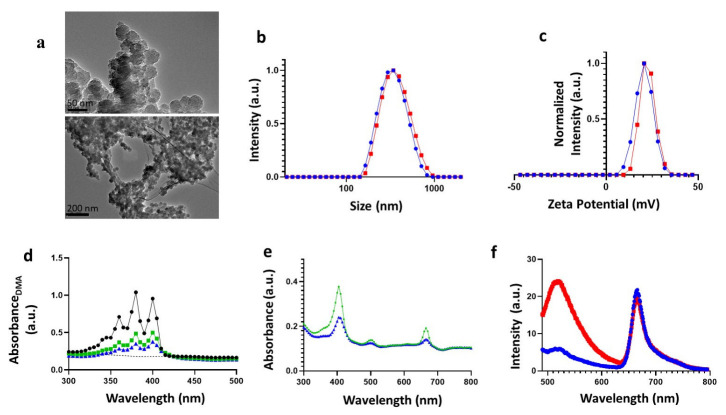
(**a**) TEM images of Ce6-PSilQ (top) and Dp44mT-Ce6-PSilQ NPs (bottom). (**b**) Hydrod-ynamic diameter of Ce6-PSilQ (blue) and Dp44mT-Ce6-PSilQ (red) NPs as measured by DLS. (**c**) ξ-potential of Ce6-PSilQ (blue) and Dp44mT-Ce6-PSilQ (red) NPs. (**d**) Indirect determination of singlet oxygen formation using DMA (black) for Ce6 (green) and Ce6-PSilQ NPs (blue). (**e**) UV-vis spectra of Ce6 (green) and Ce6-PSilQ NPs (blue). (**f**) Fluorescence spectra of Ce6-PSilQ (blue) and Dp44mT-Ce6-PSilQ (red) NPs.

In this work, we fabricated Ce6-PSilQ NPs, Dp44mT-Ce6-PSilQ NPs, and siRNA-Ce6-PSilQ NPs. These materials produce cancer cell death through the simultaneous combination of PDT and the inhibition of the pro-survival autophagy mechanism. First, we synthesized “in situ” the Ce6-silane precursor (Ce6-TES) through a conjugation reaction between the Ce6 molecule and APTES. A reverse microemulsion approach was used for the fabrication of Ce6-PSilQ NPs using Ce6-TES. TEM images of the nanoparticles were analyzed to obtain the size and morphology of Ce6-PSilQ NPs, which are spherical with a diameter of 42.3 ± 7.1 nm (*n* = 50) (Figure 1a, top). The hydrodynamic diameter of Ce6-PSilQ NPs in PBS was determined to be 324.2 ± 2.9 nm (PDI = 0.17 ± 0.02) (Figure 1b and Table S1). The ξ-potential was measured as 20.9 ± 0.4 mV (Figure 1c and Table S1). Ce6-PSilQ NPs were characterized for the number of surface-accessible amines using the ninhydrin test and measured as 1876 ± 234 nmol NH_2_/mg. The loading capacity of Ce6 in Ce6-PSilQ NPs was determined as 17.0 ± 1.4% *w*/*w* by UHPLC. Singlet oxygen generation by Ce6-PSilQ NPs after irradiation was confirmed by the drop in the absorption intensity of DMA, as seen in Figure 1d. The UV-vis absorption spectra of Ce6-PSilQ NPs are similar to those of the parent Ce6, showing maximum absorption peaks at 405 and 670 nm, which are characteristic of chlorin compounds (Figure 1e) [37].

In the case of Dp44mT-Ce6-PSilQ material, Dp44mT was added to the microemulsion during the first step before addition of the catalyst to maximize the loading of the molecule. To synthesize Dp44mT-Ce6-PSilQ NPs, the ratio 1:6 mol of Dp44mT:Ce6 was selected as the mixing ratio to be added to the reverse microemulsion during the formation of nanoparticles. The mixing ratio used is in the synergistic range of combination between Dp44mT and Ce6 for PDT, as seen in Table S2 (ESI). The TEM images were analyzed to obtain the size and morphology of Dp44mT-Ce6-PSilQ NPs, which are spherical with a diameter of 46.9 ± 8.9 nm (*n* = 50) (Figure 1a, bottom). The hydrodynamic diameter of Dp44mT-Ce6-PSilQ NPs in PBS was determined to be 376.1 ± 7.6 nm (Figure 1b and Table S1). The ξ-potential was measured as +21.8 ± 0.9 mV (Figure 1c and Table S1). The loading capacities of Dp44mT and Ce6 in Dp44mT-Ce6-PSilQ NPs were quantified as 1.2 ± 0.2 and 15.8 ± 1.6% *w*/*w* (1:6 mol ratio) using reverse-phase HPLC. Fluorescence spectroscopy further confirmed the presence of both Ce6 and Dp44mT, as observed in Figure 1f, with maximum peaks at 670 and 510 nm, respectively.

The siRNA- and sip62-Ce6-PSilQ NPs were prepared by taking advantage of the positive charge on the surface of the Ce6-PSilQ nanoparticles to electrostatically interact with siRNA [28]. Due to the small amount of material (<50 nM) obtained through this method, no further characterization of the nanoparticles was carried out.

### 3.2. In vitro Uptake of Ce6-PSilQ Nanoparticles

**Figure 2:**
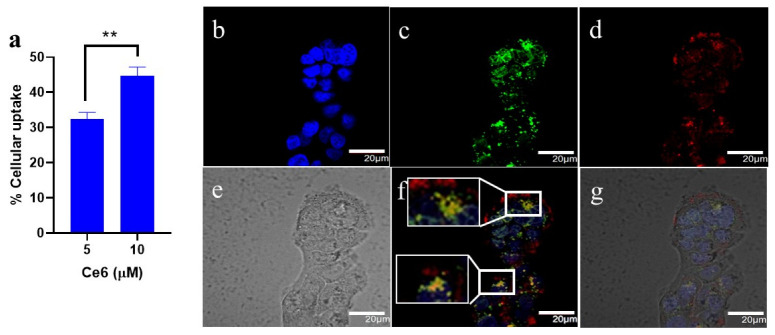
(**a**) Cellular uptake of Ce6-PSilQ NPs by HT29 cells. Confocal microscopy images of HT29 cells treated with Ce6-PSilQ NPs for (**b**) blue (Hoescht 33342) channel, (**c**) green (Lysotracker) channel, (**d**) red (Ce6-PSilQ NPs) channel, (**e**) bright channel, (**f**) merged image of blue, green, and red channels, inserts indicate spots where NPs are co-localized with lysosomes, and (**g**) merged image of all the channels. Scale bar measures 20 µm. ** *p* ≤ 0.01.

The cellular uptake of Ce6-PSilQ NPs was analyzed using flow cytometry (Figure 2a). The uptake efficiency of the particles was obtained as 33.0 ± 1.8% Ce6-positive cells at 5 µM [Ce6]. Average uptake efficiency increased by 12% when the concentration was increased to 10 µM [Ce6]. Confocal fluorescence imaging of Ce6-PSilQ NPs at 10 µM [Ce6] by HT29 cells revealed successful uptake of the nanoparticles at 37 °C after 12 h of incubation (Figure 2b–g). The overlapping fluorescence between stained lysosomes (green) and Ce6-PSilQ NPs (red) in Figure 2f depicts co-localization of the NPs in lysosomes, as indicated by the yellow spots, presumably by the endolysosomal pathway [38]. Ce6-PSilQ NPs can also be seen in the perinuclear space and cell membrane of HT29 cells.

### 3.3. Phototoxicity of Ce6-PSilQ NPs and Dp44mT-Ce6-PSilQ NPs in HT29 Cells

**Figure 3:**
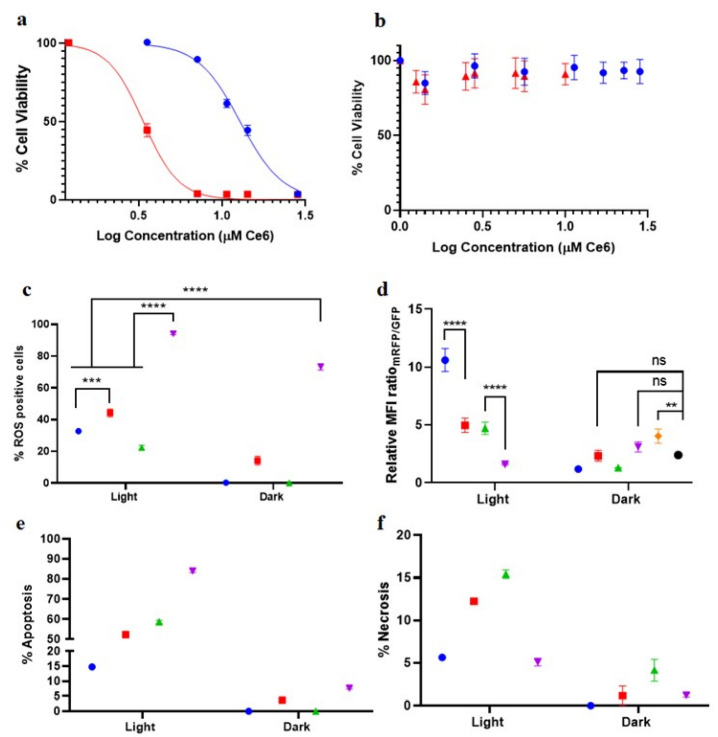
(**a**) Phototoxicity and (**b**) cytotoxicity of Ce6-PSilQ (blue) and Dp44mT-Ce6-PSilQ (red) NPs in HT29 cells. (**c**) ROS generation in Ce6-PSilQ (blue), Dp44mT-Ce6-PSilQ (red), Ce6 (green), and Ce6/Dp44mT (purple) treated HT29 cells detected by DCFH-DA ROS probe. (**d**) Autophagy flux, (**e**) apoptosis, and (**f**) necrosis analysis of HT29 cells treated with Ce6-PSilQ NPs (blue), Dp44mT-Ce6-PSilQ (red) NPs, Ce6 (green), Dp44mT/Ce6 (purple), serum starved (orange), and chloroquine (black). Data are represented as mean ± SD. Statistics: two-way ANOVA using Tukey’s multiple comparison test. **** *p* ≤ 0.0001, *** *p* ≤ 0.001, ** *p* ≤ 0.01, and ns: *p* > 0.05.

The phototoxic profile of Ce6-PSilQ and Dp44mT-Ce6-PSilQ NPs in HT29 cells was measured using an MTS assay (Figure 3a). The IC_50_ values for Ce6-PSilQ and Dp44mT-Ce6-PSilQ NPs post-irradiation were obtained as 12.8 ± 0.7 µM [Ce6] and 3.3 ± 0.2 µM [Ce6]/0.5 ± 0.0 µM [Dp44mT], respectively. More than 90% viability was observed in HT29 cells treated with Ce6-PSilQ NPs at 28 µM [Ce6] and Dp44mT-Ce6-PSilQ NPs at 25 µM [Ce6]/4 µM [Dp44mT] in the absence of light (Figure 3b). A five-fold reduction in the phototoxicity of Ce6 was observed in HT29 cells when delivered as Ce6-PSilQ NPs as opposed to free Ce6 (Table S2). The results also show an approximately four-fold decrease in the IC_50_ associated with Ce6 as a result of the combination with Dp44mT delivered to HT29 cells as a nanoformulation (Dp44mT-Ce6-PSilQ NPs) compared to Ce6-PSilQ NPs alone. Interestingly, the cytotoxicity related to Dp44mT is reduced when it is loaded to the Ce6-PSilQ NPs, as observed in the dark toxicity profile of Dp44mT-Ce6-PSilQ NPs. This can be gathered from the IC_50_ concentration of free Dp44mT (~0.1 µM) (Table S2), while Dp44mT-Ce6-PSilQ NPs did not exhibit any cytotoxicity in HT29 cells (with >90% viability) for a concentration range of 0.2–1 µM [Dp44mT] (Figure 3b).

### 3.4. ROS Production Related to Ce6-PSilQ and Dp44mT-Ce6-PSilQ Nanoparticles

Production of ROS (type I) associated with PDT was measured in HT29 cells using DCFH-DA as a fluorescent ROS probe. DCFH-DA is deacetylated by cellular esterases to a non-fluorescent compound, which is later oxidized by ROS into the fluorescent compound 2′,7′- dichlorofluorescein (DCF) [10,28]. HT29 cells were treated with Ce6-PSilQ or Dp44mT-Ce6-PSilQ nanoparticles at 2.4 µM [Ce6]. Quantification of ROS generated after irradiation (630 nm, 25 mW/cm^2^, 20 min) shows 32.8 ± 0.3% and 44.3 ± 2.5% of DCF-positive cells for the Ce6-PSilQ and Dp44mT-Ce6-PSilQ NPs, respectively (Figure 3c). In the case of free drugs, 22.4 ± 1.6% and 94.2 ± 0.8% of DCF-positive cells were measured post-irradiation for Ce6 and the physical mixture of Ce6/Dp44mT, respectively (Figure 3c). No significant ROS generation was observed for Ce6 and Ce6-PSilQ NPs in dark conditions (Figure 3c). However, in the case of Dp44mT-Ce6-PSilQ NPs and the mixture of Ce6/Dp44mT, 14.3 ± 2.6% and 73.2 ± 2.1% DCF-positive cells were observed, respectively. These results show that Dp44mT alone also has a major impact on the generation of ROS.

### 3.5. Effect of the Combination Dp44mT-Ce6-PSilQ Nanoparticles on Autophagy Inhibition

HT29 cells were modified to express mRFP GFP LC3, which allows the measuring of the autophagic flux [39]. This probe makes it possible to distinguish autophagosomes (GFP-positive and RFP-positive LC3 punctae, which are yellow) from the more acidic autolysosomes (GFP-negative and RFP-positive LC3 punctae, which are red). Autophagy flux was indirectly estimated by RFP to GFP mean fluorescence intensity (MFI) ratio of 10,000 single HT29 cells for each sample by flow cytometry. Basal flux of untreated transduced HT29 cells was used as the baseline for normalization. As seen in Figure 3d, the normalized autophagy flux of Ce6-PSilQ NPs was 10.61 ± 0.98, which was reduced to 4.98 ± 0.62 for Dp44mT-Ce6-PSilQ NPs. Cells treated with 30 µM of chloroquine were used as the positive control group for the assay expressing an MFI ratio of 2.4 ± 0.03 [40]. The decrease in autophagy flux associated with Dp44mT-Ce6-PSilQ NPs when compared to Ce6-PSilQ NPs confirms the flux inhibitory role of Dp44mT in cellular autophagy. Reduction in autophagy flux was also observed for the free drugs treatment from 4.72 ± 0.53 for free Ce6 to 1.57 ± 0.06 when combined with Dp44mT (Figure 3d). The autophagy flux of serum-starved cells was measured as 4.05 ± 0.44, which was decreased by CQ to 2.40 ± 0.01 (Figure 3d). MFI ratios of unirradiated Ce6-PSilQ and Ce6 were detected in the range of 1.1–1.3, while Dp44mT-Ce6-PSilQ and Ce6/Dp44mT were in the range of 2.3–3.1. ROS generated by Dp44mT in both Dp44mT Ce6-PSilQ and Ce6/Dp44mT mixtures contribute to autophagy above basal levels. These results were further confirmed by confocal microscopy (Figures S1 and S2).

### 3.6. Effect of the Combination Dp44mT-Ce6-PSilQ Nanoparticles on the PDT-Associated Cell Death Pathways

The amount of apoptotic and/or necrotic cells produced due to the PDT effect of Ce6-PSilQ and Dp44mT-Ce6-PSilQ NPs was analyzed by flow cytometry using the SYTOX Blue dead-cell nuclear stain assay and Annexin V Apoptosis detection kit. The concentrations of nanoparticles and free drugs evaluated in this experiment were 2.4 µM of Ce6 and 0.4 µM of Dp44mT. Cells treated with Ce6-PSilQ and Dp44mT-Ce6-PSilQ NPs in the presence of light (633 nm, 25 mW/cm^2^, 20 min) showed 14.8 ± 1.0% and 52.3 ± 0.4% of Annexin-V-positive cells, respectively (Figure 3e and Figure S3). Dark controls for Ce6-PSilQ and Dp44mT-Ce6-PSilQ NPs, under the same concentrations, were used as negative controls showing less than 1.0% and 3.7 ± 0.4% of Annexin-V-positive cells, respectively (Figure 3e). HT29 cells treated with Ce6-PSilQ and Dp44mT-Ce6-PSilQ NPs in the presence of light showed 5.6 ± 0.2% and 12.2 ± 0.3% necrotic-positive cells, respectively (Figure 3f). In the absence of light, the treatment with these nanoparticles led to less than 1% necrotic-positive cells (Figure 3f). A similar apoptotic/necrotic analysis was performed for free drugs. As depicted in Figure 3e, HT29 cells treated with Ce6 or the combination Ce6/Dp44mT after light irradiation exhibited 58.7 ± 0.8% and 84.0 ± 0.8% of Annexin-V-positive cells, respectively. Dark controls for the same free drugs show less than 1 ± 0.4% and 7.7 ± 0.4% of Annexin-V-positive cells (Figure 3e). The percentages of HT29 cells undergoing necrosis under the treatment with Ce6 and the combination of Ce6/Dp44mT in presence of light were 15.4 ± 0.5% and 5.1 ± 0.4% of necrotic-positive cells (Figure 3f). Dark controls of the same free drugs showed 4.2 ± 1.3% and 1.2 ± 0.2%. ROS from Dp44mT alone (Figure 3c) in the unirradiated samples does not cause significant cell death (Figure 3e). This indicates a co-dependent interaction between Ce6, Dp44mT, and light for an improved therapeutic outcome.

### 3.7. Effect of the Combination sip62-Ce6-PSilQ Nanoparticles on Autophagy Inhibition, Phototoxicity, and on the PDT-Associated Cell Death Pathways

**Figure 4:**
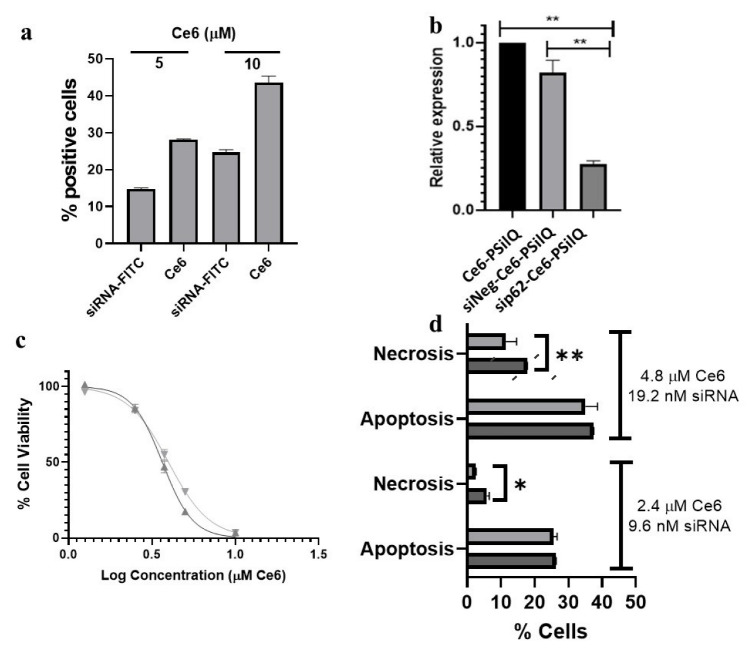
(**a**) Cellular uptake of sip62-Ce6-PSilQ NPs in HT29 cells recorded in siRNA (FITC) and Ce6 (red) channels. (**b**) Silencing of p62 gene in HT29 cells after treatment with Ce6-PSilQ NPs (black), siNeg-Ce6-PSilQ NPs (light grey), and sip62-Ce6-PSilQ NPs (dark grey). (**c**) Phototoxicity of siNeg-Ce6-PSilQ NPs (down triangles, light grey) and sip62-Ce6-PSilQ NPs (up triangles, dark grey) in HT29 cells. (**d**) Apoptosis/necrosis analysis of HT29 cells treated with siNeg-Ce6-PSilQ NPs (horizontal bars, light grey) and sip62-Ce6-PSilQ NPs (dark grey) at different concentrations. Data are represented as mean ± SD. Statistics: two-way ANOVA using Tukey’s multiple comparison test. ** *p* ≤ 0.01, * *p* ≤ 0.05.

We evaluated the performance of sip62-Ce6-PSilQ NPs in inhibiting autophagy by siRNA-mediated silencing of the p62 gene (sip62) and its impact on the PDT outcome of the nanoparticles in HT29 cells. First, we determined the siRNA cellular uptake efficiency using FITC-labeled RNA that does not have a silencing effect (siNeg^FITC^). HT29 cells were treated with siNeg^FITC^-Ce6-PSilQ NPs at 5 µM and 10 µM [Ce6] complexed to 40 nM and 20 nM siNeg^FITC^, respectively. The results showed concentration-dependent uptake of siNeg-Ce6-PSilQ (Figure 4a and Figure S4). Moreover, 28.2 ± 0.14% and 43.6 ± 1.7% of Ce6-positive cells were observed for the lower and higher concentrations. Correspondingly, 14.8 ± 0.3% and 24.8 ± 0.6% of siRNA internalization was observed for the same concentrations. No significant change was observed in the overall cellular uptake due to surface modification of Ce6-PSilQ NPs by siRNA coating.

siNeg-Ce6-PSilQ NPs were used as a negative control platform to account for any non-target specific effects of siRNA delivery. Gene silencing by sip62 was confirmed using quantitative RT-PCR and ΔΔCq analysis. The relative p62 mRNA expression normalized to Ce6-PSilQ NPs shows a significant difference for sip62-Ce6-PSilQ NPs (0.27 ± 0.03) compared to siNeg-Ce6-PSilQ NPs (0.82 ± 0.10) (* *p* < 0.01.) (Figure 4b) as an indication of the silencing of the p62 gene and most likely the p62/SQSTM1 protein.

The phototoxic effect on HT29 cells treated with sip62-Ce6-PSilQ NPs was analyzed using an MTS assay (Figure 4c) under light irradiation (633 nm, 25 mW/cm^2^, 20 min). The IC_50_ values determined from the concentration–response curve for the sip62-Ce6-PSilQ, siNeg-Ce6-PSilQ, and Ce6-PSilQ NPs are 10.3 ± 0.4 µM, 11.3 ± 0.5 µM, and 12.8 ± 0.7 µM [Ce6], respectively. A trend is observed in these IC_50_ values with the following increasing order of phototoxicity: sip62-Ce6-PSilQ > siNeg-Ce6-PSilQ > Ce6-PSilQ NPs. Nevertheless, no significant difference was found between the IC_50_ values.

The analysis of the cell death mechanisms shows that sip62- and siNeg-Ce6-PSilQ NPs afforded 26.3 ± 0.1% and 25.6 ± 0.7% of Annexin-V-positive cells with concentrations of at 2.4 µM [Ce6] and 9.6 nM [siRNA] in the presence of light (633 nm, 25 mW/cm^2^, 20 min) (Figure 4d and Figure S5). At higher concentrations, 4.8 µM [Ce6] and 19.2 nM [siRNA], sip62- and siNeg-Ce6-PSilQ NPs produced 37.4 ± 0.1% and 35.0 ± 2.6% of Annexin-V-positive cells, respectively. No significant change in apoptosis was observed due to the combination of p62 silencing and PDT. The analysis of necrosis associated with the treatment using sip62- and siNeg-Ce6-PSilQ NPs afforded 5.6 ± 0.1% and 2.6 ± 0.2% of necrotic-positive cells, respectively, at concentrations of 2.4 µM [Ce6] and 9.6 nM [siRNA]. At the higher concentrations, 4.8 µM [Ce6] and 19.2 nM [siRNA], sip62- and siNeg-Ce6-PSilQ NPs produced 17.8 ± 0.1% and 11.5 ± 2.2% of necrotic-positive cells. In this case, a trend of slightly higher numbers of necrotic cells was found for the treatments with sip62-Ce6-PSilQ NPs (*p* < 0.05).

## 4. Discussion

Photodynamic therapy triggers different types of cell death mechanisms with the most common being apoptosis and necrosis [5,10,41]. In addition to cancer cell death induced by PDT, intrinsic detoxification mechanisms to combat photooxidative stress are also activated [42]. One such adaptive response to PDT-mediated extrinsic stress is autophagy [43,44]. The high reactivity of photogenerated ROS leads to selective autophagy to remove oxidatively damaged organelles and biomolecules. Alternately, HIF-1α is another key player that confers adaptability to hypoxia, which might lead to PDT resistance by promoting the expression of the vacuole membrane protein 1 (VMP1), a protein capable of inducing the formation of autophagosomes [44]. Autophagy was found to protect PDT-treated cells from oxidative damage triggered by various PSs, such as 5-ALA, hypercin, PhotofrinTM, protoporphyrin IX, and verteporfin [45,46,47]. Protective autophagy can be repressed through pharmacological agents such as Bafilomycin-A1, Chloroquine, 3-Methyladenine, or Wortmannin [42,47]. In addition, genetic intervention is another alternative to target autophagy-related genes such as ATG3, ATG5, or Beclin-1 [48]. Moreover, regulators such as CHOP can be used as a combination strategy to quell PDT-resistance in tumor cells [49]. Although there are some reports supporting autophagy-associated cell death in PDT [50], in this work, we investigated the effect of nanoparticle-mediated combination therapy involving PDT and negative regulation of autophagy. The complexity of autophagy and its numerous steps allow for several possibilities of therapeutic intervention [44]. Two promising approaches have been explored in this study: the use of Dp44mT-Ce6-PSilQ NPs, which carry an autophagy inhibitor, and sip62-Ce6-PSilQ NPs that target the synthesis of p62/SQSTM1 autophagosome cargo protein.

In this study, we synthesized Ce6-PSilQ nanoparticles starting with the “in situ” synthesis of Ce6-silane precursor that is subsequently condensed into nanoparticles in a base-catalyzed reverse microemulsion system [27,28,31]. The nanoparticles obtained were spherical and with a diameter of 42.3 ± 7.1 nm (Figure 1a). As reported previously, PSilQ nanomaterials are distinguished for reaching a high loading capacity of the therapeutic agents [24,28,29]. In this case, the loading of Ce6 to PSilQ nanoparticles was determined to be 17.0 ± 4.0% wt. There were no significant differences found in size, morphology, hydrodynamic diameter, surface charge, and overall cargo loading capacity between Ce6-PSilQ and Dp44mT-Ce6-PSilQ NPs (Figure 1a–c and Table S1). The reduced absorption efficiency of Ce6 molecules immobilized in Ce6-PSilQ NPs compared to free solvated Ce6 directly impacts ^1^O_2_ production (Figure 1d,e). This phenomenon has been noticed for several conjugated forms of Ce6 and other PSs [10,28,51].

We evaluated the in vitro properties of the Ce6-PSilQ nanoparticles in a human colorectal cancer (HT29) cell line. Several studies have shown promising results supporting the efficacy of PDT to treat colon cancer as an adjuvant therapy at different stages of the disease [36,47]. Flow cytometry and confocal microscopy showed that HT29 cells internalized the nanoparticles effectively (>30% at 17 µg/mL) (Figure 2). The co-localization of Ce6-PSilQ NPs in the lysosomes as can be seen from the overlapping fluorescence of Ce6 (red) and LysoTracker Green in the confocal microscopy images (Figure 2f) is indicative that these nanoparticles are internalized through the endolysosomal pathway [27,28].

The phototoxic performance of Ce6-PSilQ NPs in HT29 cells showed the typical dose–response profile associated with PDT (Figure 3a). In comparison with the parent PS agent, the phototoxicity of the Ce6-PSilQ nanoparticles (IC_50_ = 12.8 ± 0.7 µM) is reduced 5-fold (Table S2). The reduction of the PDT effect is due to the encapsulation of PSs in PSilQ NPs [10]. This difference is explained by the self-quenching effect of closely packed PSs in the nanoparticles, which directly impacts the generation of ^1^O_2_ [10,30,31]. Nevertheless, the encapsulation of PSs in PSilQ NPs has major advantages for the PDT application of this platform in vivo [27]. To confirm that ROS are involved in the PDT effect, we used DCFH-DA to measure the presence of ROS such as hydroxyl, peroxyl radicals, and hydrogen peroxide in HT29 cells [52]. Flow cytometry data show that Ce6-PSilQ NPs generated a 1.5-fold higher number of DCF-positive cells than the parent Ce6 (Figure 3c). This result may be contradictory to the IC_50_ obtained for the nanoparticles and Ce6. It is important to point out that the phototoxicity of Ce6 has been mainly related to the generation of ^1^O_2_ and its localization in specific organelles, such as mitochondria [6,53,54,55]. Therefore, our results of higher generation of type I ROS by the Ce6-PSilQ NPs do not necessarily imply better phototoxicity. The analysis of the apoptosis and necrosis triggered by Ce6-PSilQ NPs and Ce6 show interesting differences in their cell death mechanism. Ce6 produced 4- and 3-fold higher numbers of apoptotic and necrotic cells, respectively, than Ce6-PSilQ NPs (*p* < 0.0001) (Figure 3e,f). The direct impact of PDT-generated ROS on mitochondria and the subsequent release of cytochrome *c* is reported as the major checkpoint controlling the induction of apoptosis [56]. Encapsulation of Ce6 in PSilQ NPs may prevent co-localization of the PS with mitochondria. Nevertheless, lysosomal membrane permeabilization (LMP) has been shown to initiate cell death by the release of cathepsins and other hydrolases into the cytosol [57,58]. Cell death by LMP might assume either apoptotic or necrotic mechanisms, depending on the occurrence of caspase activation. Therefore, we hypothesized that LMP plays an important role in the phototoxicity of Ce6-PSilQ NPs, as has been demonstrated for other platforms [59].

Autophagy is described as a catabolic mechanism characterized by vesicles engulfing dysfunctional cellular components for degradation and recycling in lysosomes [16]. High autophagy flux implies a rapid rate of recycling of carbonylated proteins and damaged organelles [60]. Autophagy is often observed as a consequence of excess intracellular ROS or oxidative stress. Previous reports have shown that premature termination of active autophagy enhanced the toxic effect of ROS in cancer cells [43,61,62]. In this work, we used Ce6-PSilQ NPs as a multifunctional platform to combine PDT together with two different strategies that target autophagy. We used a thiosemicarbazone-based autophagy inhibitor (Dp44mT), which targets the formation of autolysosome by preventing the fusion of lysosomes with autophagosomes [32]. This particular cellular process was investigated using a commercially available tandem sensor (Premo^TM^ (Milan, Italy) Tandem Autophagy sensor RFP-GFP-LC3 kit) that has the ability to monitor the various stages of autophagy through LC3B protein localization [63]. The sensor is a baculoviral construct that encodes an acid-sensitive GFP with an acid-insensitive RFP. The changes in pH due to the fusion of autophagosomes (neutral pH) with lysosomes (acidic pH) can be visualized by quantifying the loss of GFP as compared to RFP fluorescence intensity (autophagy flux) [64]. In this study, as a parameter for quantification, we measured the ratio of red to green mean fluorescence intensity of the transduced samples after PDT treatment using flow cytometry (Figure 3d). The autophagy flux of HT29 cells treated with Ce6-PSilQ NPs was reduced by 47% after treatment with Dp44mT-Ce6-PSilQ NPs as an indication of the inhibiting effect of Dp44mT on autophagy (*p* ≤ 0.0001). More importantly, the PDT outcome of Dp44mT-Ce6-PSilQ NPs showed an increase in phototoxicity, as demonstrated by the four-fold decrease in the IC50 value as compared to Ce6-PSilQ NPs alone (Figure 3a). Apoptosis analysis also provides confirmation on the enhancement of PDT effect due to the combined approach (Figure 3e). Dp44mT-Ce6-PSilQ NPs afforded 3.5-fold more apoptotic-positive cells than Ce6-PSilQ NPs. A similar trend was observed for the free drugs. Necrosis analysis showed a two-fold increase in the number of necrotic-positive cells associated with Dp44mT-Ce6-PSilQ NPs in comparison with Ce6-PSilQ NPs (Figure 3f). Our data demonstrate that by co-encapsulating the autophagy inhibitor with Ce6 in the PSilQ platform, an improved phototherapeutic effect is achieved against human colorectal cancer cells. In addition, the use of the nanoparticles to carry Dp44mT also has a major impact on reducing its cytotoxic effect (Table S2) [65], as can be seen from the decrease in ROS- and apoptotic-positive cells related to Dp44mT as compared to Dp44mT-Ce6-PSilQ NPs under dark conditions (Figure 3c,e). A major advantage of using nanoplatforms for drug delivery is their ability to reduce side effects associated with anticancer drugs [66,67].

Our second approach to inhibit the autophagy mechanism targets the p62/SQSTM1 autophagosome cargo protein by using siRNA [33]. The sip62-Ce6-PSilQ NPs efficiently reduced the expression of the p62 gene in HT29 cells (Figure 4b). Nevertheless, this silencing effect was not fully reflected in the phototherapeutic outcome of sip62-Ce6-PSilQ NPs against HT29 cells (Figure 4c). Only a minimal reduction in the IC_50_ values was observed for this material compared with the control experiments. The apoptosis analysis did not show much difference either (Figure 4d). This can be explained by compensatory mechanisms in autophagy, where cells recruit other autophagosome-specific receptors such as NBR1 and NDP52 for binding and subsequent sequestration of PDT-induced poly-ubiquitinated products of oxidation into autophagosomes for degradation upon fusion with lysosomes [54]. Similar results were reported for the shRNA-mediated knockdown of ATG5, which only partially blocked autophagic response, resulting in a marginal improvement of Hela and MCF-7 cell sensitivity to PDT [43]. Interestingly, inhibition of p62 revealed a relatively higher fraction (*p* < 0.05) of non-apoptotic cell death, which aligns with observations by other groups, where sip62 increased non-apoptotic cell death in multiple carcinoma cells in a siRNA concentration-dependent manner [33].

## 5. Conclusions

In this study, we designed, synthesized, and characterized multifunctional PSilQ nanoparticles to carry Ce6 as a PS agent and an autophagy inhibitor agent. We independently targeted the autophagy pathway at two different stages: at the early stage using sip62 or at the late stage with Dp44mT. Our results show that despite the efficient silencing of the p62 gene, which is associated with the p62/SQSTM1 autophagosome cargo protein, the final phototherapeutic outcome produced by sip62-Ce6-PSilQ NPs was not statistically different from the control experiments. The lack of phototoxic effect is most likely due to compensatory mechanisms in autophagy at the early stage to overcome the effect of PDT. Recent reports have shown that the complete knockout of ATG5 utilizing CRISPR/Cas9 genome in HeLa cells resulted in a significant increase in PDT-mediated toxicity [43]. This is a strategy worthy of exploration in the future using this platform. Our second approach, which relies on Dp44mT that targets the late stage of the autophagy mechanism, produced an additive interaction between Ce6 and Dp44mT. Our results for the autophagy flux, phototoxicity, and apoptosis/necrosis analyses demonstrated that Dp44mT-Ce6-PSilQ NPs efficiently eliminated HT29 cells with the combined performance of the photosensitizer and the autophagy inhibitor. It is also important to point out that the encapsulation of the Dp44mT molecule inside the PSilQ platform reduced its cytotoxic effect related to ROS generation without decreasing its inhibitory capability. Overall, our study demonstrated that the use of a multifunctional PSilQ system for the codelivery of a PS agent and autophagy inhibitor enhances photodynamic therapy against cancer cells. We envision that this approach can be combined with other therapies, such as chemotherapy immunotherapy or photothermal therapy, to further improve the use of PDT for the treatment of cancer.

## Data Availability

The data presented in this study are available in this article and Supplementary Materials.

## Supplementary Materials

The following supporting information can be downloaded at https://www.mdpi.com/article/10.3390/pharmaceutics15051548/s1, Extended Materials and Methods sections; Characterization of Ce6-PSilQ NPs; Figure S1. Confocal image of autophagy flux observed in Ce6-PSilQ NPs after irradiation; Figure S2. Confocal image of autophagy flux observed in Dp44mT-Ce6-PSilQ NPs after irradiation; Figure S3. Annexin/PI histograms for free Ce6, free Ce6/Dp44mT, Ce6-PSilq NPs and Dp44mT-Ce6-PSilQ NPs after irradiation.; Figure S4. Confocal image of siNeg^FITC^-Ce6-PSilQ NPs uptake in HT29 cells; Figure S5. Annexin/PI histograms for sip62-Ce6-PSilQ NPs and siNeg-Ce6-PSilQ NPs at two different concentrations after irradiation; Table S1. Structural properties of Ce6-PSilQ and Ce6-Dp44mT-PSilQ NPs; and Table S2. Tabulated IC_50_ and CI values observed in HT29 cells upon treatment with various molar ratios of Ce6:Dp44mT.
