# Supplementary material for: Autophagy Regulation Using Multimodal Chlorin e6-Loaded Polysilsesquioxane Nanoparticles to Improve Photodynamic Therapy
Source: Pharmaceutics. 2023 May 20;15(5):1548. doi: 10.3390/pharmaceutics15051548 (PMC10222236; doi:10.3390/pharmaceutics15051548)
Supplement: Supplementary file 1 [file pharmaceutics-15-01548-s001.zip › pharmaceutics-2382879-supplementary.pdf]

Supplementary Information
Autophagy Regulation Using Multimodal Chlorin e6-Loaded Polysilsesquioxane Nanoparticles to Improve Photodynamic Therapy

Materials and Methods

Chlorin e6 (Ce6), Triton X-100 (TX-100), 2',7'-dichlorodihydrofluorescein diacetate (DCFDA), PremoTM Tandem Autophagy sensor RFP-GFP-LC3 kit, LysotrackerTM Green DND-26 and SYTOXTM Blue dead cell stain were obtained from Thermo Fischer Scientific. 1- ethyl-3-(3-dimethylaminopropyl) carbodiimide (EDC) was purchased from Oakwood Chemical. Di-2-pyridylketone 4,4-dimethyl-3-thiosemicarbazone (Dp44mT), 1-hexanol and the rest of the chemicals used in this work were obtained from Sigma-Aldrich and used without any further purification unless specified otherwise. A homemade LED device emitting at 630 nm (24.5 mW/cm^2) was used for our in vitro experiments (Laboratory of Technological Support, São Carlos Institute of Physics, Brazil). A microplate reader Multiskan FC (Fisher scientific) was used for the MTS assay. Roswell Park Memorial Institute (RPMI 1640), Dulbecco Modified Eagle Medium (DMEM), penicillin-streptomycin (pen-strep), Dulbecco's phosphate buffer saline (DPBS, 1X), and trypsin were purchased from Corning. CellTiter 96® Aqueous Assay was obtained from Promega (Madison, WI, USA). Glutamax was purchased from Gibco and non-essential amino acids (NEAA) was purchased from Quality biologicals. Fetal bovine serum (FBS) was purchased from Atlanta Biologicals. Hoechst 33342 dye was purchased from Life Technologies. Sterile-filtered DMSO was used for all cell experiments and purchased from Sigma. BD Pharmingen™ Annexin V-FITC Apoptosis Detection Kit was purchased from BD Biosciences. SQSTM1/p62 siRNA (h), Control siRNA-A, Control siRNA (FITC Conjugate)-A and siRNA dilution buffer were purchased from Santa Cruz Biotechnology. PureLink RNA Mini Kit and DNAse I (RNAase Free) were purchased from ThermoFisher Scientific. iScriptTM cDNA Synthesis kit, PrimePCRTM SYBR Green Assay: SQSTM1 (h) and PrimePCRTM SYBR Green Assay:

GADPH (h) and iTaqTM Universal SYBR Green mix were purchased from Bio-Rad Laboratories USA.

Characterization of Ce6-PSilQ NPs

Hydrodynamic diameter and ζ-potential

To determine the hydrodynamic size and ζ-potential of Ce6-PSilQ NPs, the nanoparticles were dispersed under ultrasonication for 10 min in complete DMEM at a concentration of 0.1 mg/mL. The resultant dispersion was incubated at room temperature for 30 min before analysis using a Malvern Zetasizer nano. Measurements were conducted at 20 °C with a 2 min equilibration step between each subsequent measurement.

Determination of the amines chemically accessible on the surface of Ce6-PSilQ NPs

The number of surface accessible amines for Ce6-PSilQ NPs were obtained by Kaiser's Ninhydrin test. Briefly, 1 mg of Ce6-PSilQ NPs were dispersed in 1 mL of ethanol by ultrasonication at room temperature for 15 min. This dispersion was added to 4 mL of ninhydrin stock solution prepared at a concentration of 15 mg/mL (84 mM). The reaction mixture was stirred for 14 h at room temperature and subsequently centrifuged at 13000 rpm for 15 min. Supernatants from the samples were collected and analyzed for absorbance at 570 nm. A standard curve was used to determine the number of amines (-NH2) (nmol/mg) in Ce6-PSilQ NPs.

Determination of the amount loaded of Ce6 and Dp44mT

Loading amounts of Ce6 and Dp44mT in the PSilQ NPs were analyzed using Thermo scientific UHPLC plus focused series LC/UV Vanquish diode array detector system. Supernatants (5 mL) were collected from the synthesis of Ce6-PSilQ or Dp44mT-Ce6-PSilQ NPs. The supernatants were concentrated at least 10-fold by rotary evaporation. A 10 μL aliquot of the concentrated stock was subsequently diluted in 10% ethanol. A 5 μL analyte was loaded on to a Waters Symmetry Shield RP18 100 A°, 5 μm, 2.1 mm x 150 mm column. The mobile phase comprised of water and 0.1% formic acid as the aqueous phase and acetonitrile with 0.1% formic acid as the organic phase. The flow rate of the mobile phase was 0.25 mL/min. Analytes were eluted from the column starting at 10% organic phase with a linear gradient to 100% in 6 min. After a 3 min hold time at 100% organic, the column was returned to 10% organic in 4 min followed by an equilibration time of 5 min before the next analyte sample was loaded. Ce6 standards ranged from 40 to 200 ng/mL and the detection wavelength was set to 406 nm. Dp44mT standards ranged from 100 to 400 ng/mL

and the detection wavelength was set to 340 nm. Three batches were tested and the data is presented as the average ± SD.

Absorbance and Fluorescence

The absorbance and fluorescence of Ce6-PSilQ and Dp44mT-Ce6-PSilQ materials synthesized in this work were characterized by Cary 5000 UV-vis-NIR and Fluorolog spectrophotometers, respectively. The concentration of nanoparticles used in this analysis was 20 μg/mL (5.7 μM Ce6).

Determination of singlet oxygen ($^1O_2$) in solution

Singlet oxygen generation capacity of Ce6-PSilQ NPs was tested in solution using dimethyl anthracene (DMA) as the $^1O_2$ probe. The following protocol was followed, 2 mL of an aqueous solution containing 50 μM DMA and Ce6-PSilQ NPs (20 μg/mL, 5.7 μM Ce6) or free Ce6 (0.5 μM Ce6) were placed in quartz cuvettes under dark conditions. The solution was then illuminated under red light (λ = 630 nm, fluence rate = 24.5 mW/cm^2) for 10 min. The irradiated suspension of Ce6-PSilQ NPs was centrifuged at 13,000 rpm for 15 min and the supernatant was gathered for analysis. The absorption spectra of the irradiated samples were recorded. The $^1O_2$ generation was qualitatively confirmed by absorbance intensities of DMA after irradiation.

Cell culture

Human colon cancer cell line HT29 was purchased from the American Type Culture Collection (ATCC, USA). HT29 cells were cultured in Dulbecco's modified Eagle's medium (DMEM, Thermo Fisher Scientific, Waltham, MA, USA) supplemented with 10% fetal bovine serum (Thermo Fisher Scientific), 1% penicillin/streptomycin (HyClone, Logan, UT, USA), 1% Glutamax (Gibco, Grand Island, New York) and 1% non-essential amino acids (NEAA, Quality Biological, Gaithersburg, Maryland). Cells were incubated in a humidified atmosphere of 5% CO_2 at 37 °C.

Stock solutions for PDT

Ce6 and Dp44mT were dissolved in DMSO as 1 mM stock solutions respectively and stored at −20 °C, protected from light. Working solutions of Ce6 and Dp44mT were prepared fresh at various concentrations in complete DMEM before adding to cells. DCFDA was dissolved as a 10 mM stock solution in DMSO, protected from light. A 10 μM working solution of DCFDA was prepared from the 10 mM stock in serum free DMEM before adding to cells. The prepared siRNA-Ce6-PSilQ NP stock solution (100 μM Ce6, 400 nM siRNA) was serially diluted in serum and antibiotic free DMEM in the concentration range of 4-40 nM range and added to cells for treatment.

FIGURES

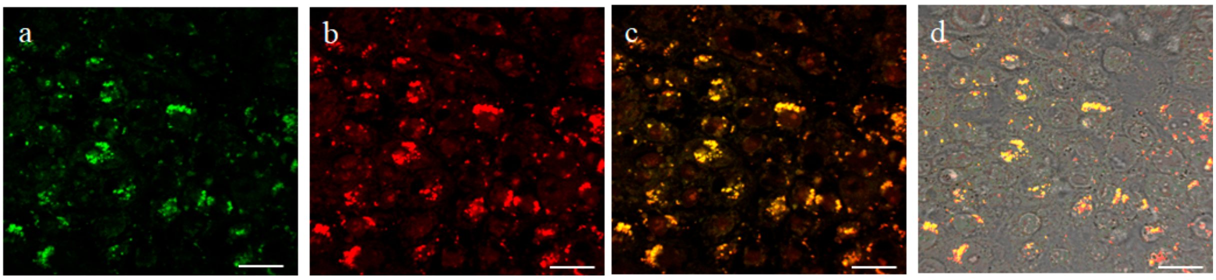

Figure S1. Confocal image of autophagy flux observed in Ce6-PSilQ NPs after irradiation. **a)** green (GFP-LC3 positive) channel, **b)** red (RFP-LC3 positive) channel, **c)** merged image of green and red channels (yellow pucta), and **d)** merged image of all the channels including bright channel. Scale bar= 20 µm.

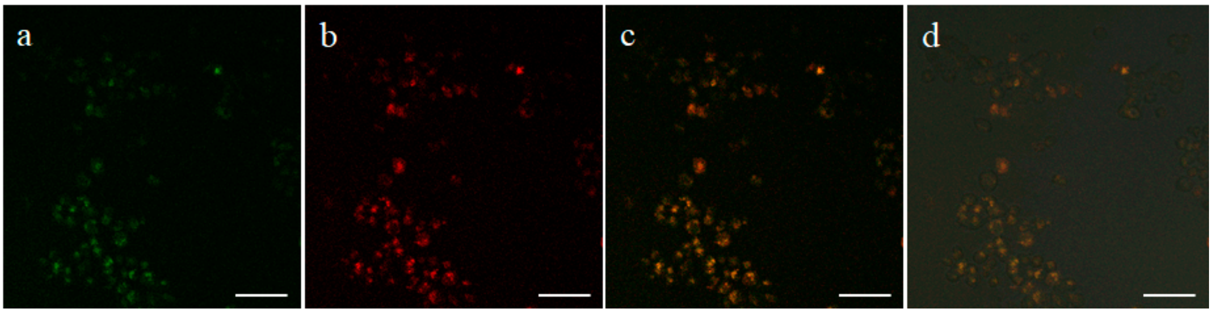

Figure S2. Confocal image of autophagy flux observed in Dp44mT-Ce6-PSilQ NPs after irradiation. **a)** green (GFP-LC3 positive) channel, **b)** red (RFP-LC3 positive) channel, **c)** merged image of green and red channels (yellow pucta), and **d)** merged image of all the channels including bright channel. Scale bar = 40 µm.

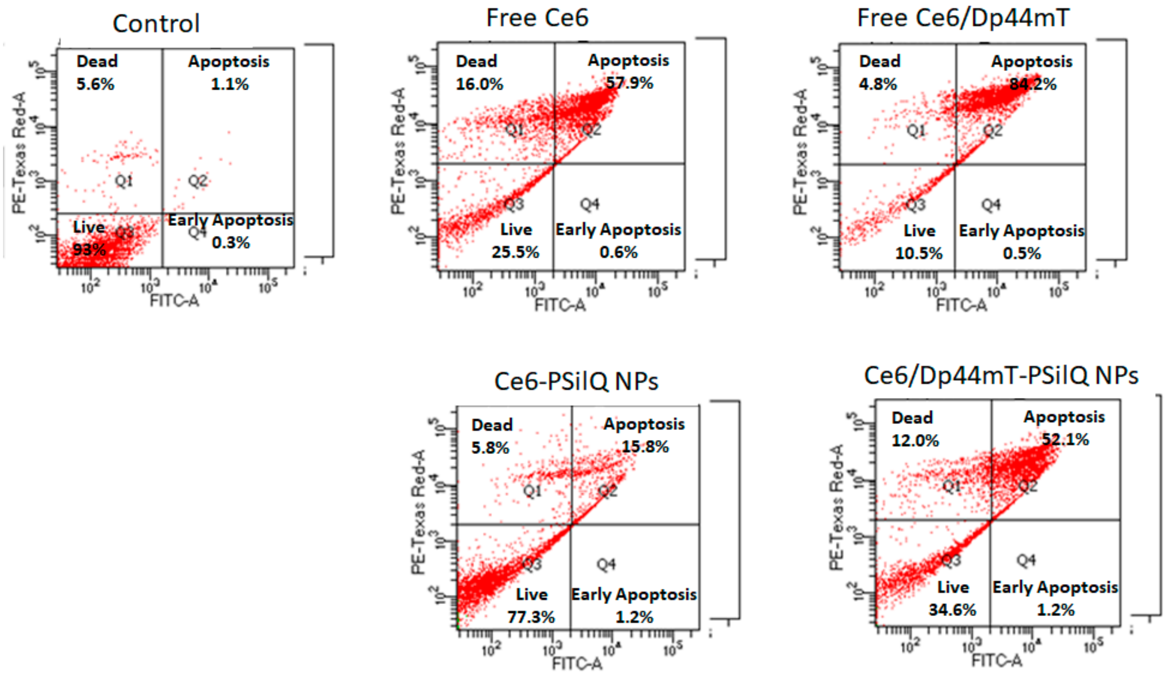

Figure S3. Annexin/PI histograms for free Ce6, free Ce6/Dp44mT, Ce6-PSilq NPs and Dp44mT-Ce6-PSilQ NPs after irradiation.

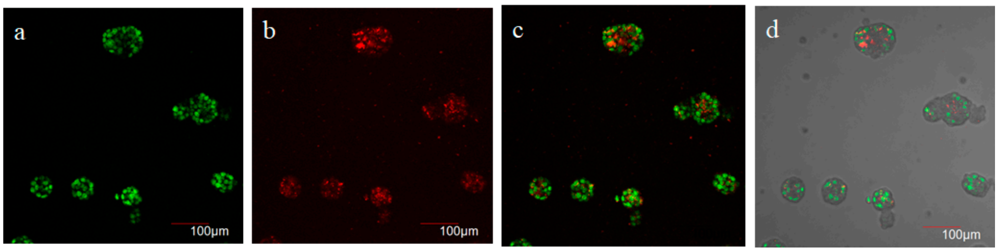

Figure S4. Confocal image of siNeg^{FITC}-Ce6-PSilQ NPs uptake in HT29 cells. **a)** green (siNeg^{FITC}) channel, b**)** red (Ce6-PSilQ NPs) channel, c**)** merged image of green and red channels, and d**)** merged image of all the channels including bright channel. Scale bar = 40 µm.

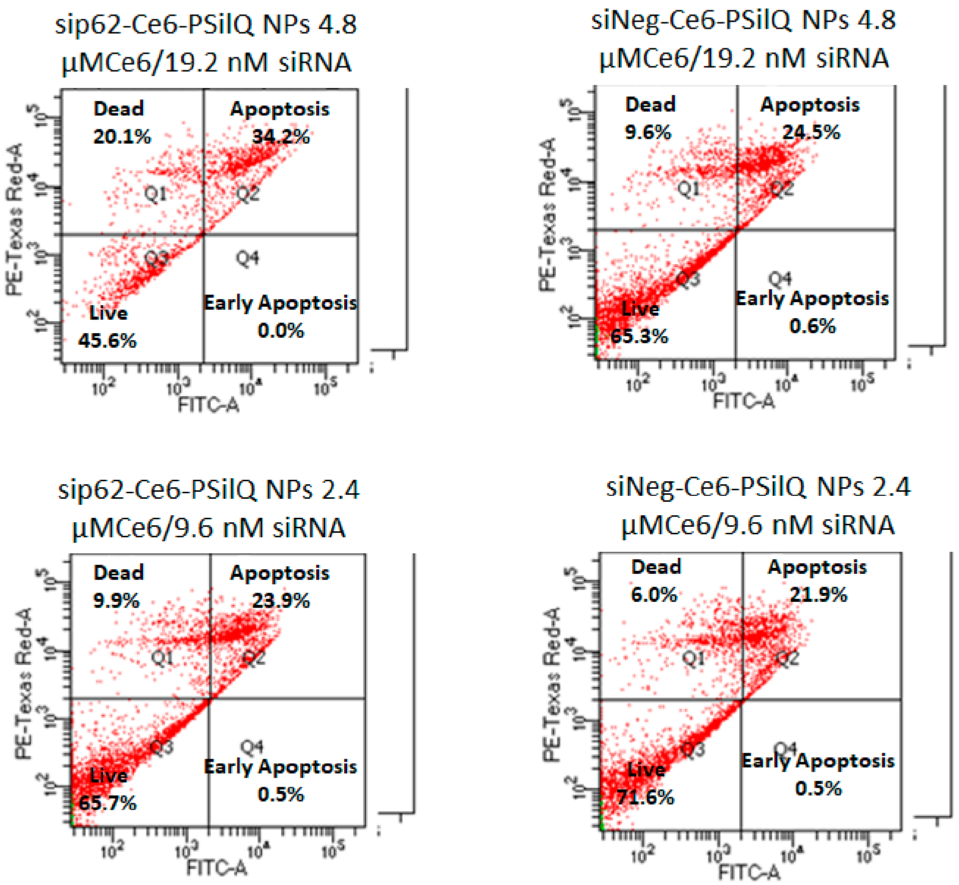

Figure S5. Annexin/PI histograms for sip62-Ce6-PSilQ NPs and siNeg-Ce6-PSilQ NPs at two different concentrations after irradiation.

TABLES

Table S1. Structural properties of Ce6-PSilQ and Ce6-Dp44mT-PSilQ NPs

	Z_{avg} (nm)	PDI	Zeta (mV)	NH_2 (nmol/ mg)
Ce6-PSilQ NPs	324.2 ± 2.9	0.17 ± 0.02	+ 20.9 ± 0.4	1876 ± 234
Ce6-Dp44mT-PSilQ NPs	376.1 ± 7.6	0.22 ± 0.02	+ 21.8 ± 0.5	NA

Table S2. Tabulated IC_{50} and CI values observed in HT29 cells upon treatment with various molar ratios of Ce6:Dp44mT.

**Ratio Dp44mT:Ce6 (μM:μM)**	**Ce6 (IC_{50} μM)**	**Dp44mT (IC_{50} μM)**	**Combination Index**
0.0375	2.486	0.016	1.124
0.1	1.115	0.021	0.647
0.225	0.696	0.034	0.620
0.6	0.221	0.055	0.652
Dp44mT	-	0.097	
Ce6	2.590		
